# Suppressing DRP1-mediated mitochondrial fission and mitophagy increases mitochondrial apoptosis of hepatocellular carcinoma cells in the setting of hypoxia

**DOI:** 10.1038/s41389-020-00251-5

**Published:** 2020-07-13

**Authors:** Xia-Hui Lin, Bai-Quan Qiu, Min Ma, Rui Zhang, Shu-Jung Hsu, Hua-Hua Liu, Jun Chen, Dong-Mei Gao, Jie-Feng Cui, Zheng-Gang Ren, Rong-Xin Chen

**Affiliations:** 1 Liver Cancer Institute, Zhongshan Hospital, Fudan University, Shanghai, China; 2 Key Laboratory of Carcinogenesis and Cancer Invasion, Ministry of Education, Shanghai, China; 3 Department of Cardiothoracic surgery, The Second Affiliated Hospital of Nanchang University, Nanchang, Jiangxi PR China

**Keywords:** Oncogenes, Autophagy

## Abstract

Transarterial embolization/transarterial chemoembolization (TAE/TACE) is the acceptable palliative treatment for hepatocellular carcinoma (HCC), mainly through ischemic necrosis induced by arterial embolization. However, how HCC cells survive under such ischemic hypoxic condition remains unclear, which can be exploited to potentiate TAE/TACE treatment. We hypothesized that targeting mitophagy can increase HCC cell apoptosis during hypoxia. HCC cells were subjected to hypoxia and then mitophagy was quantified. The role of dynamin-related protein 1 (DRP1) in hypoxia-induced HCC mitophagy was determined. Moreover, the synergistic effect of hypoxia and DRP1 inhibitor on HCC apoptosis was assessed in vitro and in vivo. Clinical association between DRP1 expression and outcome for HCC patients was validated. HCC cells that survived hypoxia showed significantly increased DRP1-mediated mitochondrial fission and mitophagy compared with cells in normoxia. Hypoxia induced mitophagy in surviving HCC cells by enhancing DRP1 expression and its translocation into the mitochondria and excessive mitochondrial fission into fragments. Blocking the DRP1 heightened the possibility of hypoxic cytotoxicity to HCC cells due to impaired mitophagy and increased the mitochondrial apoptosis, which involved decreased in mitochondrial membrane potential and mitochondrial release of apoptosis-inducing factor and cytochrome c. Additionally, DRP1 inhibitor Mdivi-1 suppressed the in vivo growth of hypoxia-exposed HCC cells. High expression of DRP1 was significantly associated with shorter survival in HCC patients. In conclusion, our results demonstrate that blocking DRP1-mediated mitochondrial fission and mitophagy increases the incidence of mitochondrial apoptosis of HCC cells during hypoxia, suggesting the new approach of targeting mitophagy to potentiate TAE/TACE.

## Introduction

In 2018, hepatocellular carcinoma (HCC) was identified as the sixth most common cancer and the fourth leading cause of cancer-related deaths worldwide, accounting for ~841,000 new cases and 782,000 deaths^1,2^. Of these, <30% patients are recommended for radical therapies (e.g., resection, liver transplantation, or ablation) and most patients are diagnosed at unresectable intermediate-advanced stage. Locoregional embolotherpies TAE/TACE (transarterial chemoembolization/transarterial embolization) as palliative treatments are commonly used for unresectable HCC^3,4^. It has been estimated that >100,000 TACE procedures are performed in China every year. TAE and TACE prolong the survival of unresectable HCC patients, mainly through inducing tumor ischemic necrosis by hepatic artery embolization^5,6^. TAE/TACE can extend survival, but the incidence of local tumor recurrence is relatively high. In excised HCC specimens, peripheral residuals of viable tumor cells are common after TAE/TACE, which may contribute to local recurrence^7^. Furthermore, emerging evidences show that HCC cells capable of surviving TAE/TACE obtain a more invasive phenotype^8^. However, how HCC cells develop an adaptation to survive the ischemic hypoxia induced by TAE/TACE is unclear. This could be analyzed to enhance the embolic effects of TAE/TACE.

Mitochondrial dynamics, such as fusion, fission, and removal, play a critical role in maintaining physiological functions of cells^9,10^. Mitophagy is a conservative defense mechanism of the selective degradation of the damaged mitochondria via the autophagic pathway, which contributes to cellular homeostasis^11^. However, cancer cells often benefit from the mitophagy process to survive by adapting to several stresses including DNA damage, inflammation, ischemia, hypoxia, and nutrient deprivation^12^. This led to the hypothesis that targeting mitophagy would disrupt the adaptive response of HCC cells to ischemic hypoxia induced by TAE/TACE, which then could enhance the embolic effects.

Mitophagy involves a series of stages: mitochondrial fission, subsequent autophagosome formation, and fusion with lysosome^9^. The dynamin-related protein 1 (DRP1) mainly regulates mitochondrial fission that is prerequisite for mitophagy^13–15^. DRP1 is recruited to the mitochondrial membrane to drive mitochondrial division, forming spirals that constrict and fragment the mitochondrion apart^16^. Since DRP1-dependent mitochondrial fission is essential for mitophagy initiation, it was considered that suppressing mitophagy could increase apoptosis of HCC cells in the adaption to hypoxia by targeting DRP1-mediated mitochondrial fission, suggesting a new potential way of strengthening TAE/TACE antitumor effect.

This study showed the following details: (i) HCC cells survived hypoxia with a significant increase in DRP1-mediated mitochondrial fission and mitophagy. (ii) Blocking DRP1 enhanced cytotoxic hypoxia to HCC cells by impairing mitophagy and increasing mitochondrial apoptosis, which included the decrease in mitochondrial membrane potential and mitochondrial release of apoptosis-inducing factor (AIF) and cytochrome c. (iii) DRP1 inhibitor Mdivi-1 suppressed the in vivo growth of hypoxia-surviving HCC cells. (iv) DRP1 was highly expressed in HCC tissues, predictive of a poor prognosis of patients. This study demonstrates that in the setting of hypoxia, blocking DRP1-mediated mitochondrial fission and mitophagy increases mitochondrial apoptosis of HCC cells, suggesting a potential approach in improving therapeutic effects of TAE/TACE.

## Results

### HCC cells survived hypoxia with a significant increase in DRP1-mediated mitochondrial fission and mitophagy

**Fig. 1:**
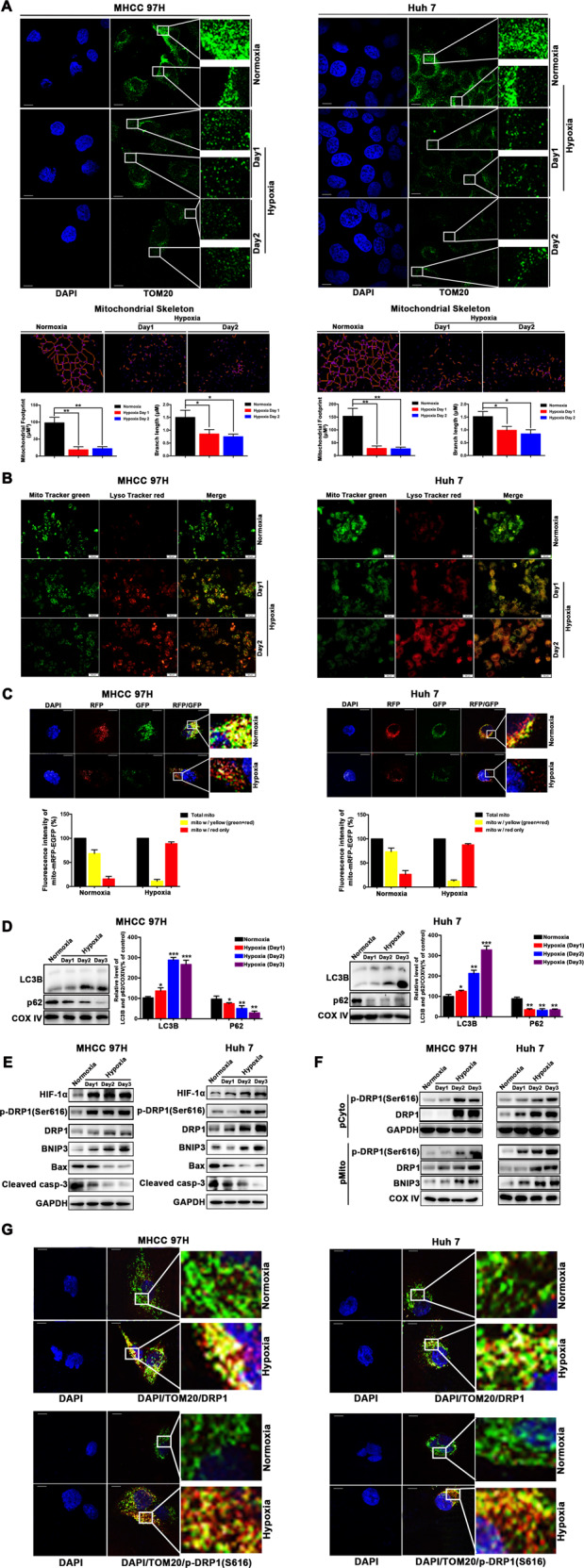
DRP1-mediated mitochondrial fission and mitophagy were activated in hypoxia-surviving HCC cells. **a** HCC cells surviving the hypoxic condition showed a distinctly fragmented mitochondrial morphology under a confocal microscopy. The mitochondrial skeleton morphologies were analyzed using an ImageJ macro tool. After exposed to hypoxia for 1 day or 2 days, MHCC97H and Huh7 cells were immunostained with specific antibody to the outer mitochondrial membrane 20 (TOM20) (green). Nuclei were counter-stained with DAPI. Scale bar 10 μM. **b** Immunofluorescence analysis showed an increase in colocalization (yellow puncta) of lysosomes (Lyso Tracker Red) and mitochondria (Mito Tracker Green). The yellow puncta indicated mitochondria-containing autolysosomes. **c** Analyses of the fluorescence signal of MHCC97H and Huh7 cells from a dual fluorescence reporter (mito-mRFP-EGFP plasmid) after exposed to hypoxia for 2 days indicated the visual analysis of mitophagic flux under a confocal microscopy (yellow color, no mitophagy; red color, mitophagy. Scale bar 10 μM. **d** LC3B and p62 protein in mitochondrial fraction in HCC cells during hypoxia was measured by western blot analysis. **e** Hypoxia-inducible factor-1α (HIF-1α), DRP1, phosphorylation (Ser616) of DRP1, BNIP3, Bax and cleaved caspase-3 (cleaved casp-3) in hypoxia-surviving HCC cells was assessed by western blot analysis. **f** DRP1, phosphorylation (Ser616) of DRP1 and BNIP3 were determined by western blot analysis of cytosolic and mitochondrial fractions (pCyto, purified cytosolic; pMito, purified mitochondria). **g** MHCC97H and Huh7 cells were exposed to hypoxia for 2 days, and confocal immunofluorescence showed mitochondrial translocation of Drp1 and phosphorylation (Ser616) of DRP1 as indicated by immunostaining with specific antibodies to DRP1 (red) or phospho-Drp1 (Ser616) (red) and TOM20 (green). Nuclei were counter-stained with DAPI. In the zoomed images, the yellow color indicates DRP1 or phospho-DRP1 (Ser616) in mitochondria. Scale bar 10 μM. *P < 0.05, **P < 0.01, ***P < 0.001.

The HCC cells that survived hypoxia showed a significant increase of mitochondrial fission and mitophagy, as indicated by the distinctly fragmented mitochondrial morphology, reduced mitochondrial footprint as well as the mean branch length analyzed using an ImageJ macro tool in the zoomed images of Fig. 1a, an increase of colocalization (yellow puncta) of lysosomes (Lyso Tracker Red) and mitochondria (Mito Tracker Green) (Fig. 1b), and an apparent increase of mitophagic flux as visual analysis (red fluorescence) by a dual fluorescence reporter under a confocal microscopy (Fig. 1c). The fluorescent staining of TOM20 or Mito Tracker Green was weaker whereas the intensity of Lyso Tracker Red was stronger in HCC cells subjected to hypoxia, indicating that an accelerated mitochondrial degradation (including mitophagy) by lysosomes is triggered during the adaption of HCC cells to hypoxia, leading to the decrease of mitochondrial mass. Furthermore, the levels of mitochondrial LC3B protein was remarkably increased and p62 protein was significantly decreased in mitochondrial fraction in HCC cells exposed to hypoxia (Fig. 1d), indicating an accelerated mitophagosome formation (an event of mitophagy). Moreover, hypoxia-treated HCC cells had a significantly lower mitochondrial mass (as measured by COX IV staining, a marker to monitor the degradation process of mitophagy) than cells in normoxia (Fig. S1). These findings indicate that mitophagy is triggered in surviving HCC cells in hypoxia.

**Fig. 2:**
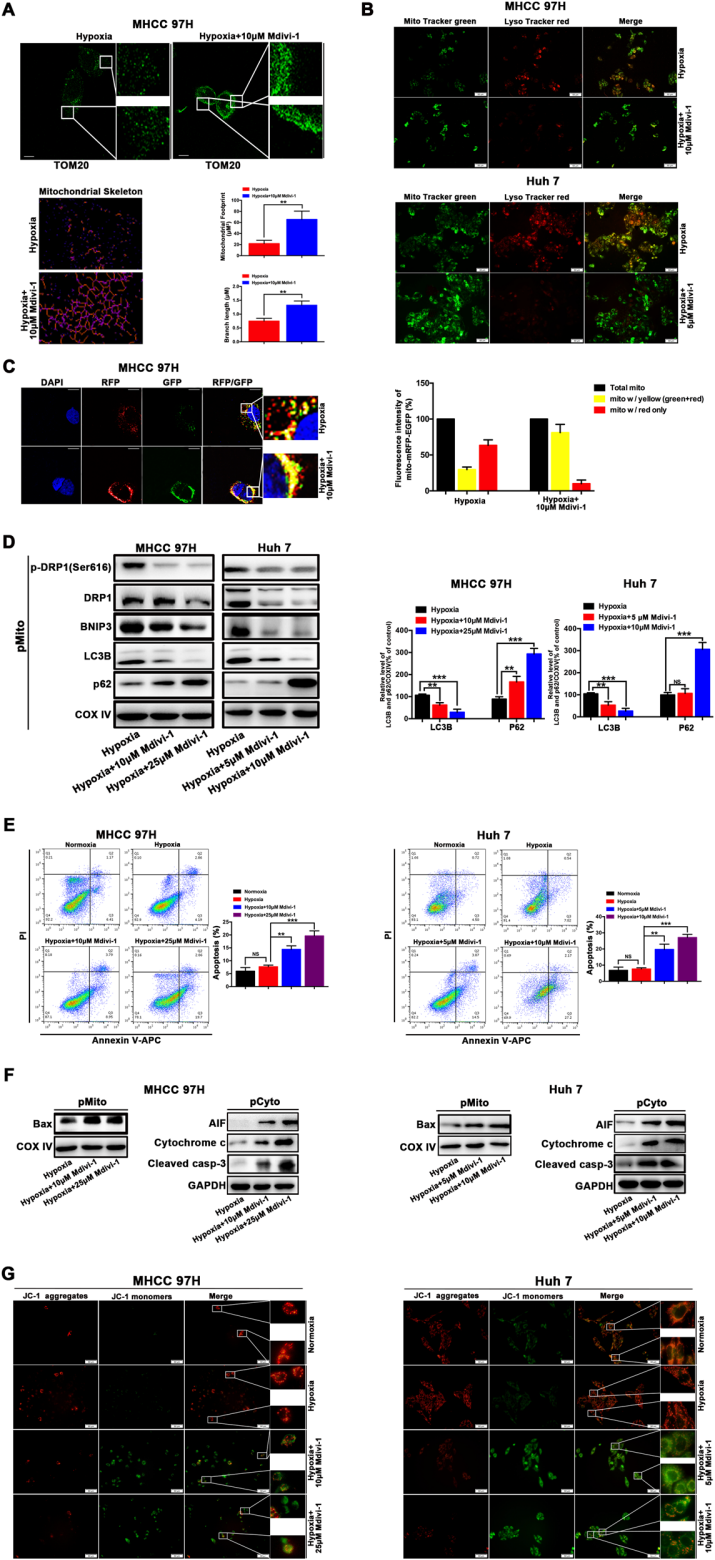
Blocking DRP1-mediated mitochondrial fission and mitophagy increased mitochondrial apoptosis of HCC cells in the setting of hypoxia. **a**–**c** Mdivi-1 suppressed the hypoxia-induced mitochondrial fission and mitophagy in HCC cells by the increased normal tubular mitochondrial morphology indicated by immunostaining with specific antibody to the outer mitochondrial membrane 20 (TOM20), reduction of yellow puncta shown by colocalization of lysosomes (Lyso Tracker Red) and mitochondria (Mito Tracker Green), and the reduced in visual analysis of mitophagic flux indicated by a dual fluorescence reporter (mito-mRFP-EGFP plasmid). Scale bar 10 μM. The mitochondrial skeleton morphologies were analyzed using an ImageJ macro tool. **d** Mdivi-1 inhibited mitochondrial translocation of DRP1 and phospho-DRP1 (Ser616), resulting in reduced BNIP3 and LC3B, and increased p62 in mitochondrial fraction, as determined by western blot analysis of mitochondrial fractions. **e** Mdivi-1 increased apoptosis of HCC cells during hypoxia as measured by flow cytometry and using Annexin V/PI staining. **f** Mdivi-1 induced the mitochondrial increase of Bax and promoted cytochrome c and apoptosis-inducing factor (AIF) release from mitochondria to cytosol, and an increase of cleaved caspase-3 (cleaved casp-3) in cytosol, as shown by western blot analysis of cytosolic and mitochondrial fractions (pCyto, purified cytosolic; pMito, purified mitochondria). **g** Mdivi-1 treatment resulted in the change of mitochondrial membrane potential as indicated by the red fluorescence and green fluorescence using JC-1 staining. Red fluorescence: JC-1 aggregates, Green fluorescence: JC-1 monomers. *P < 0.05, **P < 0.01, *** *P* < 0.001.

Given the important role of DRP1 in mitochondrial fission, DRP1 in hypoxia-induced mitophagy in surviving HCC cells was assessed. As shown in Fig. 1e, hypoxia promoted both the DRP1 expression and DRP1 phosphorylation (Ser616) in surviving HCC cells relative to the cells in normoxia. In addition, hypoxia-surviving HCC cells displayed an enhanced mitochondrial translocation of DRP1 and significant mitochondrial accumulation of phosphorylated DRP1 by western blot analysis of cytosolic and mitochondrial fractions (Fig. 1f). Additionally, surviving HCC cells in hypoxia displayed the enhanced mitochondrial translocation of Drp1 and phosphorylation (Ser616) of DRP1 (merged yellow spots) (Fig. 1g). More importantly, DRP1 inhibition by Mdivi-1 significantly suppressed hypoxia-induced mitochondrial fission and mitophagy in HCC cells, as indicated by the increased normal tubular (not fragmented) mitochondrial morphology (Fig. 2a), and a reduction of yellow puncta containing lysosome with mitochondria (Fig. 2b), and a prominent reduction in visual analysis of mitophagic flux (red fluorescence) (Fig. 2c). The fluorescence of mitochondria was stronger, and the intensity of lysosome was decreased in HCC cells in Hypoxia + Midivi-1 group, suggesting the increased mitochondria mass due to the blockage of mitochondrial degradation by mitophagy inhibitor Midivi-1 and the reduced lysosomal mass. Also, it is reported that blocking DRP1-dependent mitochondria fission can impair lysosome function^17^. In addition, LC3B was reduced whereas P62 was accumulated in surviving HCC cells in Hypoxia + Midivi-1 group (Fig. 2d), indicating that the degradation process of mitochondrial autophagic bodies is inhibited by Midivi-1. Moreover, Mdivi-1 inhibited mitochondrial translocation of phosphorylated DRP1 in surviving HCC cells in hypoxia compared to cells in normoxia (Fig. 2d). These results demonstrate that DRP1-mediated mitochondrial fission and subsequent mitophagy are significantly activated in hypoxia-surviving HCC cells.

### DRP1 and BNIP3 upregulation involved in hypoxia-induced mitophagy to attenuate apoptosis

It has been reported that DRP1-mediated mitochondrial fission is required for BNIP3-induced mitophagy in cardiac myocytes^18^. Hypoxia-inducible factor-1 (HIF-1) dependent expressions of DRP1 and phosphorylated DRP1 and mitophagy-related protein BNIP3 were significantly upregulated in hypoxia-surviving HCC cells (Fig. 1e). Similar to the DRP1-mediated mitochondrial fission activated during mitophagy as described above, BNIP3 was significantly enriched in mitochondrial fraction (Fig. 1f), suggesting that mitochondrial translocation and accumulation of BNIP3 is involved in mitophagy. Parallel to these changes, pro-apoptosis proteins Bax and cleaved caspase-3 significantly decreased in surviving HCC cells under hypoxic condition (Fig. 1e). BAX and cleaved caspase-3 was reduced in hypoxia-surviving HCC cells compared to the cells in normoxia, suggesting that hypoxia-surviving HCC cells can down-regulate apoptosis-related proteins to survive in the adaption to hypoxia. These data suggest that DRP1-mediated mitochondrial fission and BNIP3-related mitophagy is greatly activated in HCC cells during the adaption to the hypoxic environment and then attenuate the apoptosis.

### Blocking DRP1-mediated mitochondrial fission and mitophagy increased mitochondrial apoptosis of HCC cells during hypoxia

**Fig. 3:**
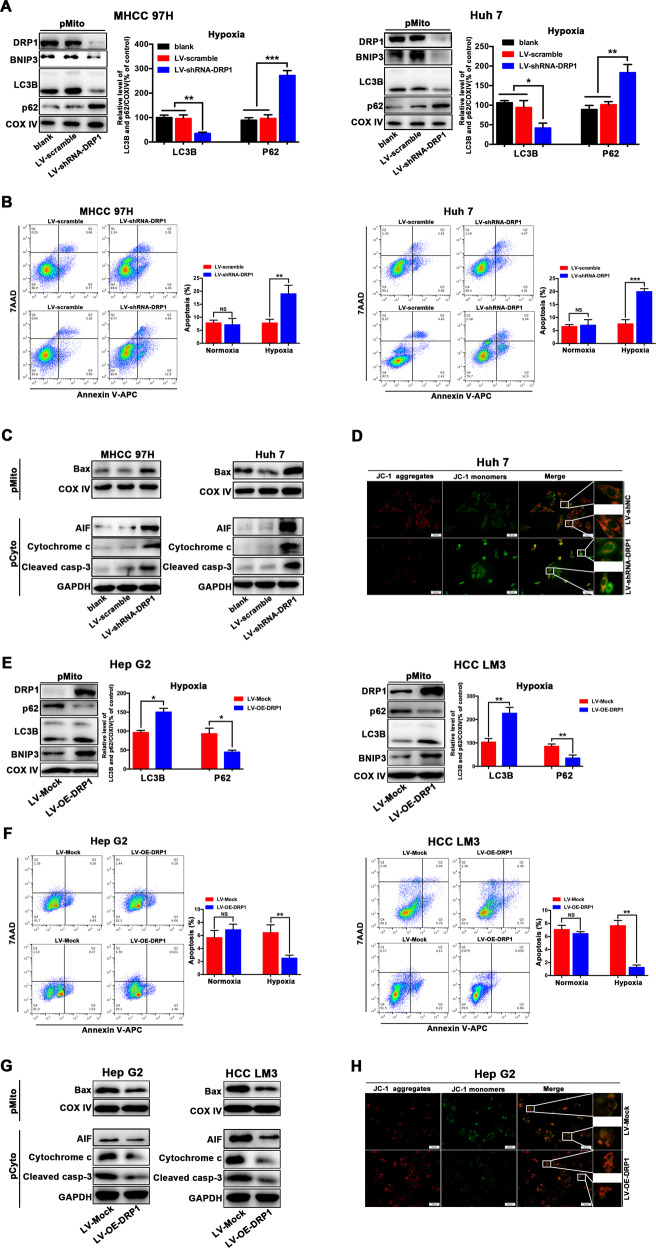
DRP1 influenced mitochondrial apoptosis of HCC cells in hypoxia. **a** DRP1 knockdown by lentiviral vectors expressing short hairpin RNA targeting DRP1 (LV-shRNA-DRP1) decreased mitophagy-related protein BNIP3 and LC3B, and increased p62 in mitochondrial fraction in hypoxic HCC cells. **b** DRP1 knockdown by LV-shRNA-DRP1 increased apoptosis of HCC cells in hypoxia, as determined by flow cytometric analyses using Annexin V-PE/7-AAD staining. **c** DRP1 knockdown by LV-shRNA-DRP1 induced the mitochondrial increase of Bax and promoted cytochrome c and apoptosis-inducing factor (AIF) release from mitochondria to cytosol, and an increase of cleaved caspase-3 (cleaved casp-3), as analyzed by western blot. **d** DRP1 knockdown resulted in mitochondrial membrane potential loss as indicated by the decreased red fluorescence and increased green fluorescence using JC-1 staining. **e** DRP1 overactivation by lentiviral vectors overexpressing DRP1 (LV-OE-DRP1) increased mitophagy-related protein BNIP3 and LC3B and decreased p62 in mitochondrial fraction in hypoxic HCC cells. **f** Upregulation of DRP1 by LV-OE-DRP1 led to the reduced apoptosis of HCC cells in hypoxia as determined by flow cytometric analyses using Annexin V-PE/7-AAD staining. **g** DRP1 overexpression by LV-OE-DRP1 resulted in reduced mitochondrial accumulation of Bax, a less cytochrome c and AIF from mitochondria, and a decreased cleaved caspase-3 (cleaved casp-3) expression. **h** DRP1 overexpression resulted in less mitochondrial membrane potential loss using JC-1 staining. *P < 0.05, **P < 0.01, ***P < 0.001.

Mitochondrial dynamics is associated with mitochondrial apoptosis^19,20^. As described above, DRP1-mediated mitochondrial fission and mitophagy were trigged in hypoxia-surviving HCC cells. DRP1 inhibition suppressed mitochondrial fission and significantly reduced mitophagy-related protein BNIP3 and LC3B and upregulated mitochondrial p62 protein in mitochondrial fraction (Fig. 2d), indicating that DRP1 inhibition may block DRP1-mediated mitochondrial fission and subsequent mitophagy, impairing hypoxia-induced mitophagy. As shown in Fig. S2, pharmacologic block of DRP1 by Mdivi-1 induced the cellular morphological changes from a long spindle-shaped morphology into round phenotype, similar to the morphology of HCC cells undergoing apoptosis. Furthermore, Mdivi-1 increased apoptosis of HCC cells in the setting of hypoxia (Fig. 2e), suggesting DRP1 inhibition renders HCC cells more sensitive to cytotoxic hypoxia. For the mechanism, DRP1 blockage induced a significant mitochondrial accumulation of Bax (Fig. 2f), a classical inducer of mitochondrial permeability transition, resulting in a massive cytochrome c and AIF release from the mitochondria to the cytosol, and an increased activation of cleaved caspase-3 (Fig. 2f), suggesting that DRP1 inhibition increases mitochondrial apoptosis of HCC cells during hypoxia. In Fig. 2f, increases in the cytosolic levels of the cytochrome c and AIF were observed, suggesting the cytosolic release of cytochrome c and AIF from mitochondria is required for apoptosis following mitophagy inhibition. It has been reported that translocation of Bax to the mitochondria alters the outer mitochondrial membrane permeability^21,22^. In Fig. 2g, mitochondrial membrane potential was not significantly decreased in HCC cells under hypoxic condition, suggesting the mitochondrial membrane potential can be compensated in hypoxic surviving HCC cells. Notably, blocking DRP1 resulted in a significant loss of mitochondrial membrane potential as indicated by the decreased red fluorescence and increased green fluorescence detected in JC-1 staining (Fig. 2g). Similar findings were obtained using genetic blockage of DRP1 by lentivirus carrying DRP1 shRNA (Fig. 3a–d). In Fig. 3c, increases in the cytosolic levels of cytochrome c and AIF were shown, suggesting the cytosolic release of cytochrome c and AIF from mitochondria is required for apoptosis following mitophagy inhibition by DRP1 knockdown. Taken together, these data indicate that blocking DRP1 enhances the cytotoxic effect of hypoxia through the impaired mitophagy and increased mitochondrial apoptosis of HCC cells, which involved the decrease in mitochondrial membrane potential and mitochondrial release of AIF and cytochrome c, an increased activation of cleaved caspase-3.

On the other hand, DRP1 overexpressing in hypoxic HCC cells (HepG2, MHCCLM3) resulted in increased mitophagy-related protein BNIP3, LC3B and decreased p62 in mitochondrial fraction (Fig. 3e), demonstrating that upregulation of DRP1 can promote mitophagy. DRP1 overexpression resulted in reduced apoptosis in hypoxia HCC cells (Fig. 3f), which resulted from decreased mitochondrial accumulation of Bax (Fig. 3g), lesser mitochondrial membrane potential loss (Fig. 3h), a lesser cytochrome c and AIF release from the mitochondria, and a decreased cleaved caspase-3 (Fig. 3g). In Fig. 3g, decrease in the cytosolic levels of cytochrome c and AIF were observed, suggesting the cytosolic release of cytochrome c and AIF from mitochondria is inhibited following mitophagy induction by DRP1 overexpression. These results indicate that DRP1 over-activation can protect hypoxia-surviving HCC cells from mitochondrial apoptosis.

### DRP1 inhibitor Mdivi-1 suppressed the in vivo growth of hypoxia-exposed HCC cells

**Fig. 4:**
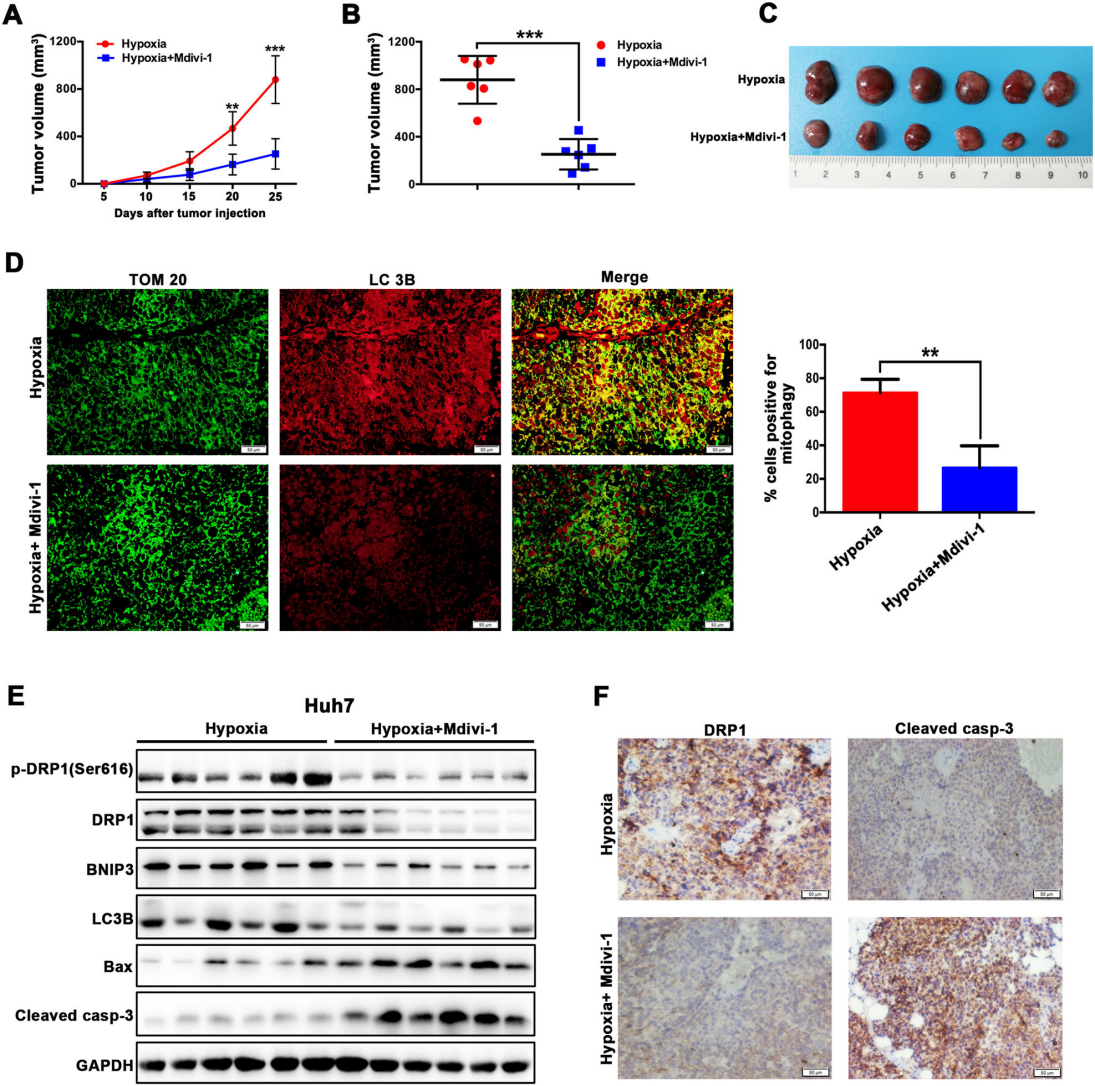
DRP1 inhibitor Mdivi-1 suppressed the in vivo growth of hypoxia-treated HCC cells. **a**–**c** Compared with the control group, Mdivi-1 administration inhibited in vivo tumor growth of hypoxic HCC cells as indicated by tumor growth rate and tumor size. **d** In the Mdivi-1 treatment group, mitophagy was significantly blocked by Mdivi-1 as indicated by immunofluorescence using colocalization with TOM20 (green) and LC3B (red). In the zoomed images, the yellow puncta indicated the mitophagy. **e** In the Mdivi-1 treatment group, the expressions of mitophagy-related proteins DRP1, phospho-DRP1 (Ser616), BNIP3, LC3B was significantly decreased and apoptotic proteins Bax and cleaved caspase-3 were markedly increased. **f** The level of DRP1 and cleaved caspase-3 were validated using immunohistochemistry staining, the result showed that DRP1 was remarkably inhibited, whereas cleaved caspase-3 was upregulated in tumors of Huh7 cells + Mdivi-1 group. *P < 0.05, **P < 0.01, ***P < 0.001.

Hypoxia-treated HCC cells were subcutaneously injected into nude mice to determine whether targeting DRP1-mediated mitophagy could promote apoptosis of hypoxic HCC cells in vivo. When compared with the control group, Mdivi-1 administration resulted in significant inhibition of tumor growth of hypoxic HCC cells as indicated by tumor growth rate and tumor size (Fig. 4a–c). In the Mdivi-1 treatment group, mitophagy was significantly blocked by DRP1 inhibitor Mdivi-1 as shown by colocalization of TOM20 and LC3B (Fig. 4d). Consistent with the apparently inhibitory effect of Mdivi-1 on tumor growth, mitophagy-related proteins DRP1, BNIP3, and LC3B expression were significantly reduced, and apoptotic proteins Bax and cleaved caspase-3 expression were significantly increased in the Mdivi-1 treatment group (Fig. 4e). Additionally, according to immunohistochemical analyses, DRP1 was remarkably inhibited, whereas cleaved caspase-3 was upregulated in tumors from Huh7 cells + Mdivi-1 group (Fig. 4f). These data suggest that DRP1 inhibitor Mdivi-1 suppresses the in vivo growth of hypoxia-exposed HCC cells through blockage of DRP1-mediated mitophagy and increased cell apoptosis.

### DRP1 upregulated in HCC tissues and correlated with poor prognosis of patients

**Fig. 5:**
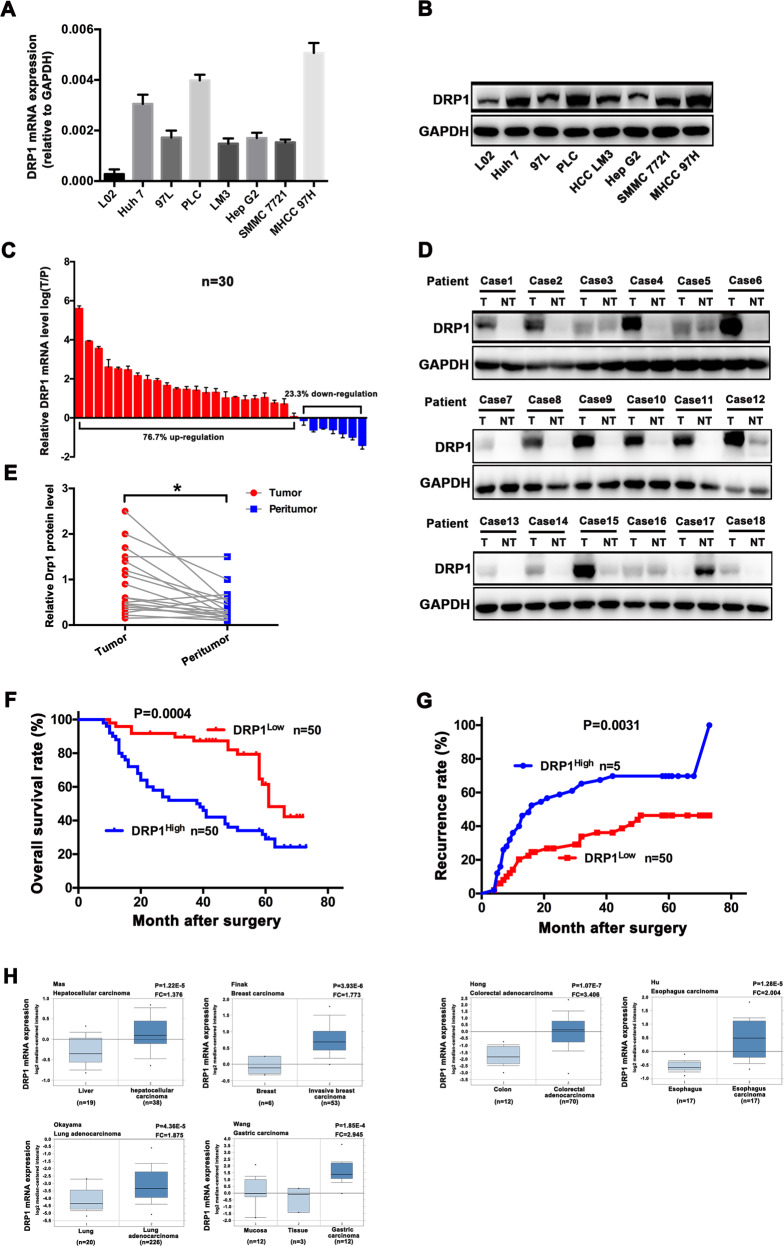
DRP1 expression was upregulated in HCC tissues and correlated with poor prognosis of HCC patients. **a**, **b** The mRNA expression and protein expression of DRP1 was assessed in seven HCC cell lines (PLC/PRF/5, HepG2, SMMC7721, MHCC97L, MHCC97H and HCCLM3) and liver cells L02, as detected by qRT-PCR and western blot analysis. **c**–**e** In 30 HCC patients, DRP1 at both the mRNA and protein levels in the HCC tissues were significantly higher than those in non-tumoral liver tissues. Representative western blot analysis of DRP1 protein expression in the HCC tissues (T) and the matched nontumor tissues (NT). **f**, **g** High expression of DRP1 was significantly associated with shorter overall survival (OS) and higher recurrence rate in HCC patients. **h** DRP1 mRNA was assessed in cancer tissues relative to the corresponding normal tissues, using datasets from the publicly available Oncomine database (www.oncomine.org). *P < 0.05, **P < 0.01, ***P < 0.001.

The mRNA and protein expression of DRP1 was significantly upregulated in seven HCC cell lines compared with liver cells L02 (Fig. 5a, b). Similarly, DRP1 at both mRNA and protein levels in human HCC tissues were remarkably higher than those in non-tumoral liver tissues from 30 HCC patients (Fig. 5c–e). Furthermore, immunohistochemistry showed that expression of DRP1 was mainly distributed in the cytoplasm of tumor cells in HCC tissues compared with corresponding peritumoral tissues (Fig. S3A). In an independent set of 100 HCC patients, DRP1 expression was noticeably upregulated in stages III–IV HCC patients (Fig. S3B). High DRP1 expression in tumor tissues was not only correlated with HCC biological aggressiveness (large tumor size, poor differentiation and late stage) (Table S1), but also associated with poor prognosis of patients (shorter OS and higher recurrence) (Fig. 5f, g). Additionally, univariate and multivariate analysis (Table S2) showed that high expression of DRP1 was an independent prognostic factor for OS (*P* = 0.001, HR = 2.449, 95% CI: 1.412–4.249), along with tumor size (*P* = 0.021, HR = 1.889, 95% CI: 1.098–3.248). Furthermore, analysis of datasets from the publicly available Oncomine database (www.oncomine.org) showed that DRP1 mRNA was not only significantly elevated in HCC tissues compared with non-tumoral liver tissues, but also markedly upregulated in many other cancer tissues relative to the corresponding normal tissues (Fig. 5h), suggesting DRP1 is overexpressed in several human tumor tissues. These results indicate that DRP1 is highly expressed in HCC tissues and is predictive of poor prognosis of HCC patients.

## Discussion

This study demonstrates that DRP1-mediated mitochondrial fission and mitophagy are activated in HCC cells during their adaption to hypoxia to attenuate apoptosis. Targeting DRP1-mediated mitochondrial fission and subsequent mitophagy increases mitochondrial apoptosis of HCC cells in the setting of hypoxia. Furthermore, this study elucidates the underlying mechanism that suppressing DRP1-mediated mitochondrial fission and mitophagy could increase Bax mitochondrial translocation, resulting in the loss of mitochondrial membrane potential, the massive release of AIF and cytochrome c from mitochondria, and triggering mitochondrial apoptosis of hypoxic HCC cells, augmenting hypoxic cytotoxicity to HCC cells. These findings have promising clinical implications in improving the TAE/TACE efficacy for HCC.

TAE/TACE, a widely used treatment for unresectable HCC, exploits the preferential blood supply of HCC from hepatic artery (>95%), mainly inducing ischemic necrosis through arterial embolization^3,5^. However, only a small proportion of treated HCC lesions show complete necrosis after TAE/TACE and most of tumor nodules contain viable HCC cells at the necrotic margin, resulting in high recurrence^23^. Hence, it is important to characterize how these HCC cells survive ischemic hypoxia to strengthen TAE/TACE efficacy and eliminate residual tumor cells.

Mitophagy is an important protective mechanism in the selective removal of mitochondria to maintain cellular homeostasis under physiopathological conditions^24^. In response to various stresses, mitophagy plays pivotal roles in several diseases, such as cancer^25^, diabetes^26^, cardiovascular diseases^27^, and neurodegenerative disorders^28^. Mitophagy is implicated in cancer treatment resistance^29,30^. This study showed that HCC cells survived hypoxia by modulation of mitochondrial dynamics to attenuate apoptosis through activation of DRP1-mediated mitochondrial fission and mitophagy.

DRP1 is essential in mitochondrial fission and mitophagy initiation. Some studies have illustrated the existence of cytosolic Ca^2+^/DRP1 or Wnt/β-catenin/DRP1 axis in mitochondrial function regulation for cancer^31–33^. Multiple posttranslational modifications of phosphorylation, ubiquitination and sumoylation regulate the functions of DRP1^34^. In this study, we provided the evidence that mitochondrial recruitment of serine 616 (S616)-phosphorylated DRP1 and mitophagy-related protein BNIP3 were responsible for mitochondrial fission and mitophagy, respectively. Hypoxia can trigger autophagy-dependent cell death via the induction of BNIP3^35,36^. BNIP3 is a specific activator of mitophagy independent of its role in apoptotic signaling^37^. It is shown that hypoxia-triggered mitophagy involved HIF-1 upregulation, DRP1-mediated mitochondrial fission and mitochondrial translocation of BNIP3. Consistent with the finding that mitophagy induced by BNIP3 requires DRP1-mediated mitochondrial fission in myocytes^18^, the results of this study underscore the importance of DRP1-mediated mitochondrial fission and subsequent BNIP3-related mitophagy for hypoxia-surviving HCC cells. Notably, disruption of DRP1 activity by pharmacologic inhibitor Mdivi-1 (an effective DRP1 inhibitor, inhibiting mitochondrial fission) or genetic knockdown suppressed mitochondrial fragmentation and hypoxia-induced mitophagy, resulting in a significant increase of mitochondrial apoptosis of hypoxic HCC cells, potentiating cytotoxic hypoxia to HCC cells. Conversely, DRP1 overactivation hindered mitochondrial apoptosis in hypoxic HCC cells. Mechanistically, DRP1 inhibition increased mitochondrial translocation of pro-apoptotic protein Bax, resulting in the loss of mitochondrial membrane potential and abundant release of AIF and cytochrome c from mitochondria to the cytosol to trigger mitochondrial apoptosis of hypoxic HCC cells, thereby enhancing hypoxic cytotoxicity to HCC cells. Therefore, interference of DRP1-induced mitochondrial fission and mitophagy leads to a considerable increase of mitochondrial apoptotic signaling in hypoxic HCC cells. Besides inhibiting DRP1 phosphorylation, Mdivi-1 down-regulated DRP1 expression in this study. As reported by other authors^32,38^, inhibition of DRP1 by Mdivi-1 decreased the DRP1 expression in their experiments. Mdivi-1 is reported to have additional targets, which may be responsible for DRP1 down-regulation. However, the mechanism needs to be elucidated.

In this study, we show that blocking DRP1-mediated mitochondrial fission and mitophagy by Mdivi-1 increases the incidence of mitochondrial apoptosis of HCC cells during the adaption to hypoxia, implicating a new potential approach of targeting mitophagy to potentiate TAE/TACE. This is consistent with the conclusions from previous studies. As reported previously, over-expression of Drp1 can promote HCC progression by enhancing the proliferation of HCC cells^2^ or facilitating tumor-associated macrophage infiltration^39^. Targeting DRP1-dependent mitochondrial fission by siRNA knockdown or treatment with Mdivi-1 can significantly induce HCC cells arrest to reduce tumor growth^2^, attenuate lipid metabolism to reduce hepatocarcinogenesis^40^, and even improve the antitumor capacity of NK cells^41^. Inhibition of mitophagy by Mdivi-1 promotes cancer cell death to anticancer agents^41,42^. Han et al. have reported that DRP1 inhibition by Mdivi-1 or DRP1 knockdown increases cisplatin sensitivity of ovarian cancer cells under hypoxia^43^. Along with our study, those evidences suggest that mitochondrial dynamics of cancer cells adapting to microenvironment could be a potential target for anticancer therapy, although some authors reported that inhibition of mitochondrial fission attenuated treatment-induced apoptosis in cancer cells^44,45^.

DRP1 overexpression has been correlated with poor prognosis of patients in many cancers. The prognostic role of DRP1 in HCC was reported in one paper^39^ and the sample size of patients (*n* = 69) was relatively small. More evidence is needed. In this study, we showed that DRP1 upregulation was associated with poor prognosis in 100 patients with HCC.

Our study has some limitations. First, although hypoxia can influence mitophagy, whether hypoxia induced mitochondrial or lysosome biogenesis, mitochondria-lysosome crosstalk and the role of the PGC-1 network in mitochondrial biogenesis is not involved in this study. Tohme et al.^46^ reported that hypoxia up-regulated mitochondrial biogenesis in hypoxic HCC cells. The increased mitochondrial density was shown in a mouse HCC cell line Hepa1–6 cells in hypoxia. In this study, hypoxia decreased the content of mitochondrial Tom20 as indicated by the weaker immunofluorescent staining in Fig. 1a, which may be caused by mitochondrial biogenesis and mitochondrial clearance. We suspected that mitochondrial degradation (mitophagy) was accelerated in the adaption of HCC cells to hypoxia because the immunofluorescent staining of Tom20 was increased in hypoxic HCC cells treated with mitophagy inhibitor Mdivi-1 in Fig. 2a. Second, the possibility that other mitophagy-related molecules such as PINK1- Parkin and NIX mediate hypoxia-induced mitophagy cannot be exclude^47^. Third, hypoxia caused alterations in the decreased oxidative phosphorylation and cytochrome c oxidase activity^48^ and the increased ROS production in mitochondria^49^. Intracellular ATP content and ROS levels are also associated with mitophagy^50,51^. However, DRP1 inhibition by Mdivi-1 had an impact on intracellular ATP content and ROS levels (Fig. S4), implying that DRP1-mediated mitophagy is essential in ATP and ROS production. Fourth, Mdivi-1 not only impairs DPR1-mediated mitochondrial fission, but also decreases mitochondrial oxidative metabolism to induce cell death^52,53^. Therefore, it is possible that Mdivi-1 may exert its pro-apoptotic effects on hypoxia-surviving HCC cells via other mechanisms. Fifth, accumulation of Bax from cytosol into the mitochondria, which triggers the release of cytochrome c and AIF, was observed following mitophagy inhibition by Midivi-1. The mechanism needs to be elucidated. Sixth, hypoxia is one of the effects after blocking blood flow to a tumor. TAE/TACE treatment not only deprives tumors of oxygen, but also restrains essential nutrients. Therefore, in vitro experiments could be performed by exposure of cells to ischemic culture condition (e.g. 1% oxygen with ischemic media including 1% FBS, and 1 mmol/L glucose) simulating TAE/TACE-like ischemia. Also, in vitro hypoxia-surviving HCC cells to simulate the residual tumor cells after TAE/TACE is suboptimal. TAE/TACE performed in a rat HCC model will be better.

**Fig. 6:**
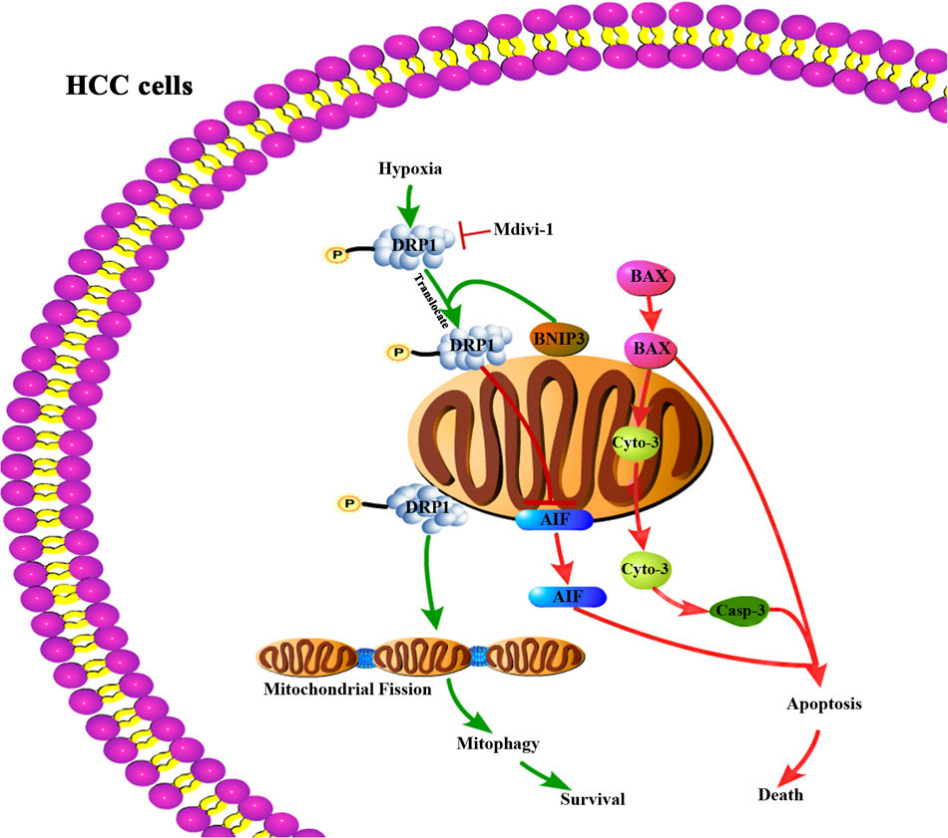
Suppressing DRP1-mediated mitophagy promotes mitochondrial apoptosis of HCC cells in the setting of hypoxia. Schematic diagram shows that DRP1-mediated mitophagy attenuates apoptosis of HCC cells in hypoxia and suppressing DRP1-mediated mitochondrial fission and mitophagy increases mitochondrial apoptosis of HCC cells.

In conclusion (Fig. 6), this study demonstrates that DRP1-mediated mitochondrial fission and mitophagy are activated in hypoxia-surviving HCC cells to attenuate apoptosis, and targeting DRP1-mediated mitochondrial fission and subsequent mitophagy increases mitochondrial apoptosis of HCC cells during hypoxia, suggesting a new approach to enhance the embolic efficacy of TAE/TACE treatment.

## Materials and methods

### Mitophagic flux

The dual fluorescence reporter plasmid pAT016 (p-mito-mRFP-EGFP, a kind gift from Dr. Andreas Till and Dr. Aleem Siddiqui, University of California) was used for visual analysis of mitophagic flux, as previously described^54^. The pAT016 encodes a mitochondrial targeting sequence fused in-frame with RFP and EGFP genes. GFP fluorescence is quenched in low pH conditions whereas RFP signal fluorescence can still be visualized in mitophagosomes and acidic mitophagolysosomes. Hence, yellow (the combination of green and red fluorescence in mitochondria) indicates normal condition, whereas the presence of RFP fluorescence alone indicates mitophagolysosomes, the fusion of the engulfed mitochondria (mitophagosomes) with lysosomes, suggesting the completion of mitophagy.

### Immunofluorescence assay

As described, the cells grown on chamber slides were transfected with the plasmid, followed by immunofluorescence assay under a confocal laser scanning microscopy (LSM510; Zeiss, Germany). Further, they were cultured with cell-permeant fluorophore MitoTracker Green (Invitrogen/Molecular Probes) or LysoTracker Red (Invitrogen/Molecular Probe) to label mitochondria and lysosomes, and were visualized under a confocal laser scanning microscopy (LSM510; Zeiss, Germany).

The cells cultured on slides were fixed with 4% paraformaldehyde, washed, permeabilized with 0.3% Triton X-100, blocked with 5% BSA, and incubated with primary antibody at 4 °C overnight, and were then incubated with the appropriate secondary antibody. The nuclei were counterstained with 4, 6-diamidino-2-phenylindole (DAPI) (Yeasen, Shanghai, China). Images were taken using a confocal laser scanning microscopy (LSM510; Zeiss, Germany) and the quantification of these images (at least 10 cells/sample) was done using ImageJ software. The mitochondrial network morphology was analyzed using an ImageJ macro tool as described previously^55^.

### Western blot

As described, protein was extracted from cells or tissues using RIPA cell lysis with Protease Inhibitor Cocktail. Mitochondrial protein extraction was done with the use of a mitochondrial isolation kit. The cells were homogenized, and the cytosolic and mitochondrial fractions were separated by Percoll gradient fractionation. Equivalent amounts of protein from each fraction were subjected to analyses. The mitochondrial fraction was isolated using the Cell Mitochondria Isolation Kit (Beyotime Biotechnology) based on the manufacturer’s instructions. In brief, cells were harvested, added with 2.5 ml mitochondrial separation reagent, and then gently suspended at 4 °C for 15 min. The cell suspension was homogenized using a glass homogenizer ten times for 10 s each, centrifuged at 600 × *g* at 4 °C for 10 min and followed by centrifugation at 11,000 × *g* at 4 °C for 10 min. The sediment was the mitochondrial fraction. The proteins were quantified by using the BCA kit, subjected to 12% SDS-PAGE for separation, and transferred to 0.45 μM PVDF membranes (Millipore, USA). Then the membranes were blocked with skimmed milk and incubated with primary antibodies at 4 °C overnight, followed by incubation with the corresponding HRP-conjugated secondary antibody (PeproTech), and the bands were visualized by enhanced chemiluminescence. The intensity of protein expression was measured using ImageJ software.

### Immunohistochemistry

As described previously, immunohistochemistry was carried out using the EnVision two-step visualization system (GeneTech, Shanghai, China). Briefly, 5μm thick sections of tumor specimens were deparaffinized with xylene, rehydrated with a graduated series of ethanol, and blocked with 3% H_2_O_2_. After antigen-retrieval using a microwave, the slides were blocked with 5% BSA and then incubated with primary antibodies against DRP1 (1:500, Abcam) at 4 °C overnight, followed by incubation with secondary antibodies and visualization with 3,3-diaminobenzidine (DAB) as a chromogen. The slides were counterstained with hematoxylin. Images were taken through a light microscope (Olympus). Immunostaining were scored by two investigators blinded to clinicopathological data and according to the staining intensity (0 = no staining, 1 = weak staining, 2 = moderate staining, 3 = strong staining) and the percentage of positive tumor cells (0 = no positive cells, 1 = 1–25% positive cells; 2 = 26–50% positive cells, 3 = 51–75% positive cells, 4 = >75% positive cells). The summed score ranged from 0 to 7 where 0 to 3 was classified as low expression level and 4 to 7 was considered as high expression level.

### Other materials and methods

For details of other materials and methods, please see Supplementary Experimental procedures file.

## Supplementary information

Supplementary Figure S1

Supplementary Figure S2

Supplementary Figure S3

Supplementary Figure S4

Supplemental Figure legends

Supplementary Table S1

Supplementary Table S2

Supplementary Experimental Procedures
