# Supplementary figures and images for: Suppressing DRP1-mediated mitochondrial fission and mitophagy increases mitochondrial apoptosis of hepatocellular carcinoma cells in the setting of hypoxia
Source: Oncogenesis. 2020 Jul 13;9(7):67. doi: 10.1038/s41389-020-00251-5 (PMC7359348; doi:10.1038/s41389-020-00251-5)

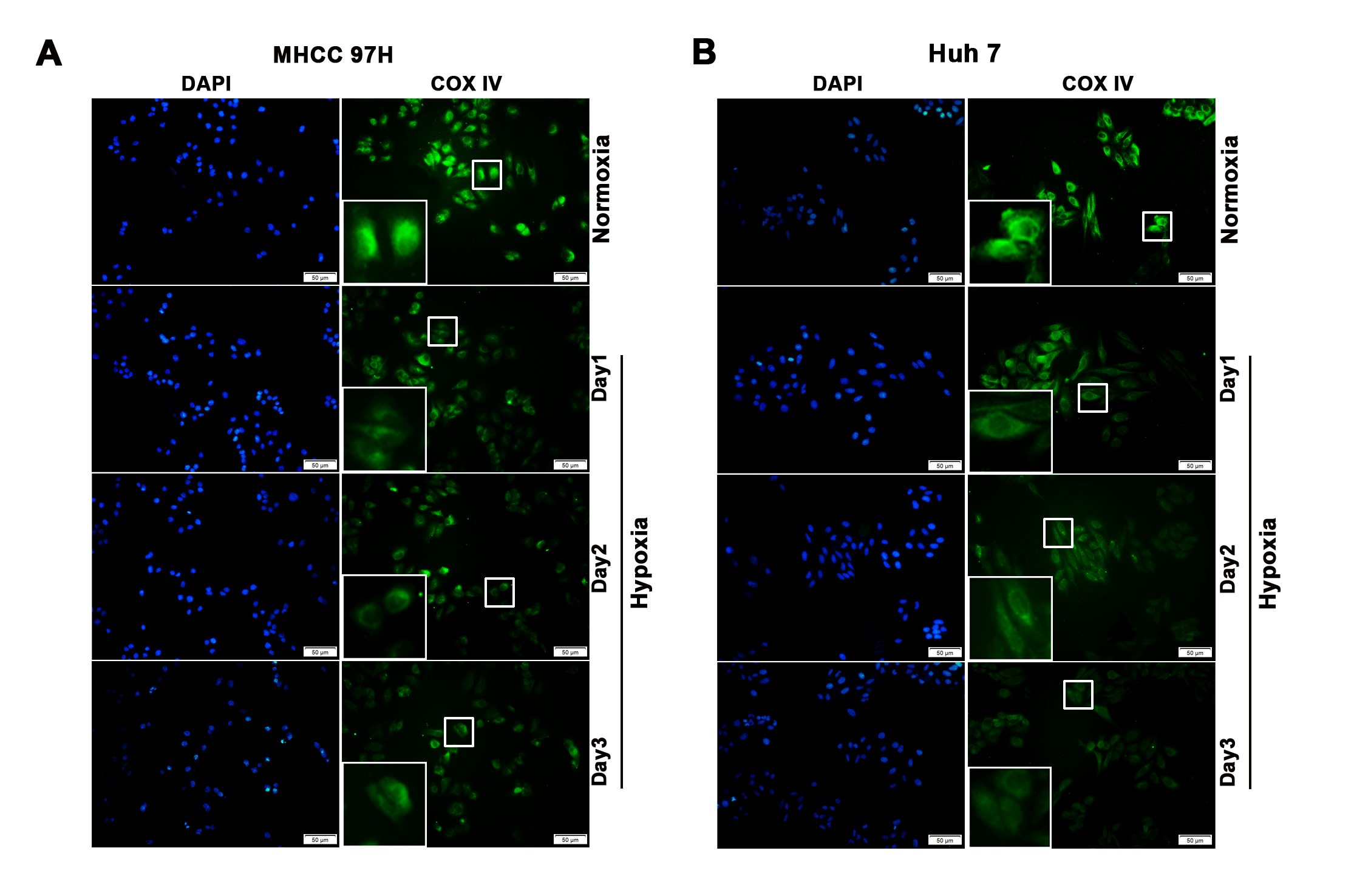

Supplement: Supplementary file 1 — Supplementary Figure S1 [file 41389_2020_251_MOESM1_ESM.tif]

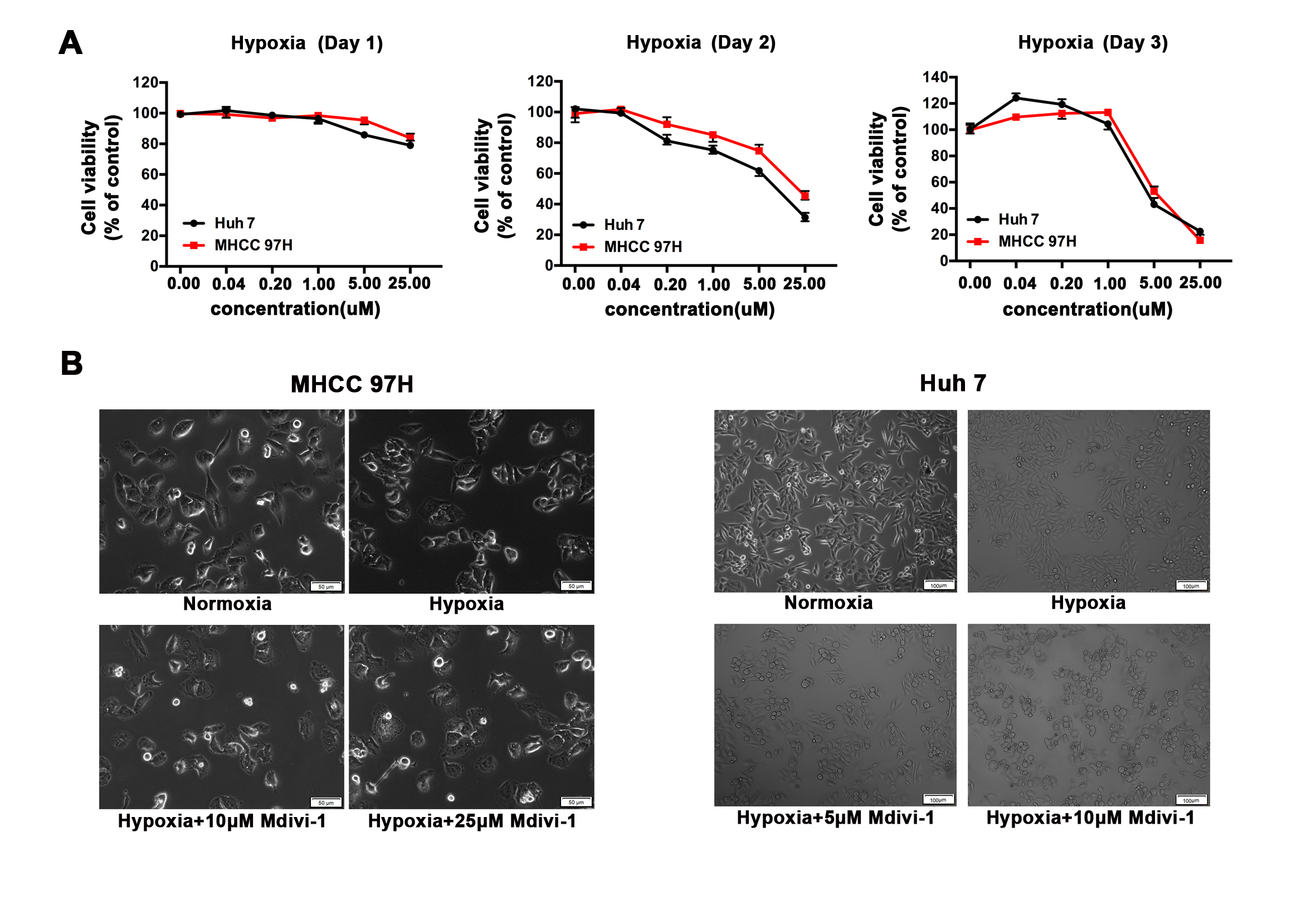

Supplement: Supplementary file 2 — Supplementary Figure S2 [file 41389_2020_251_MOESM2_ESM.tif]

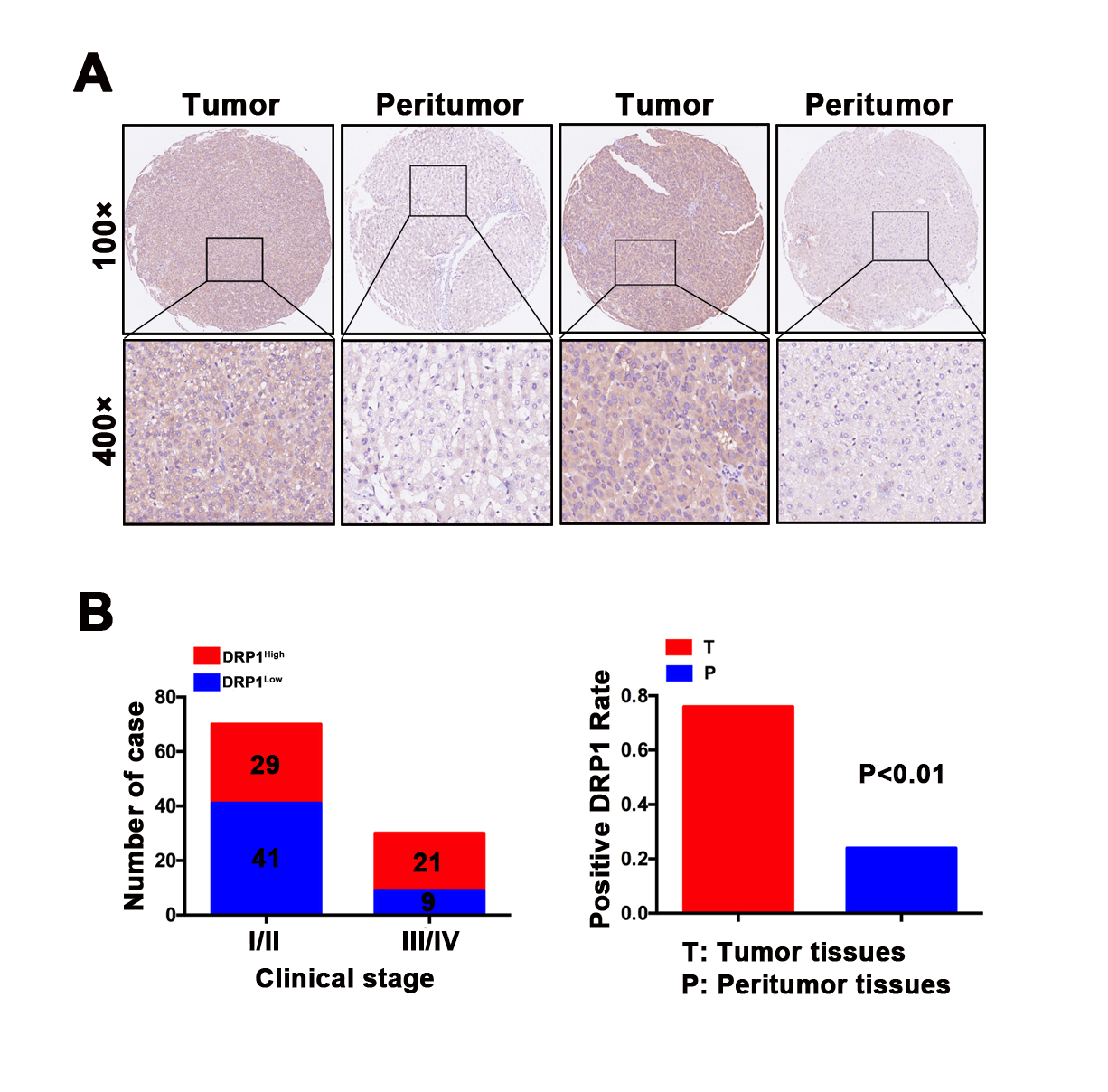

Supplement: Supplementary file 3 — Supplementary Figure S3 [file 41389_2020_251_MOESM3_ESM.tif]

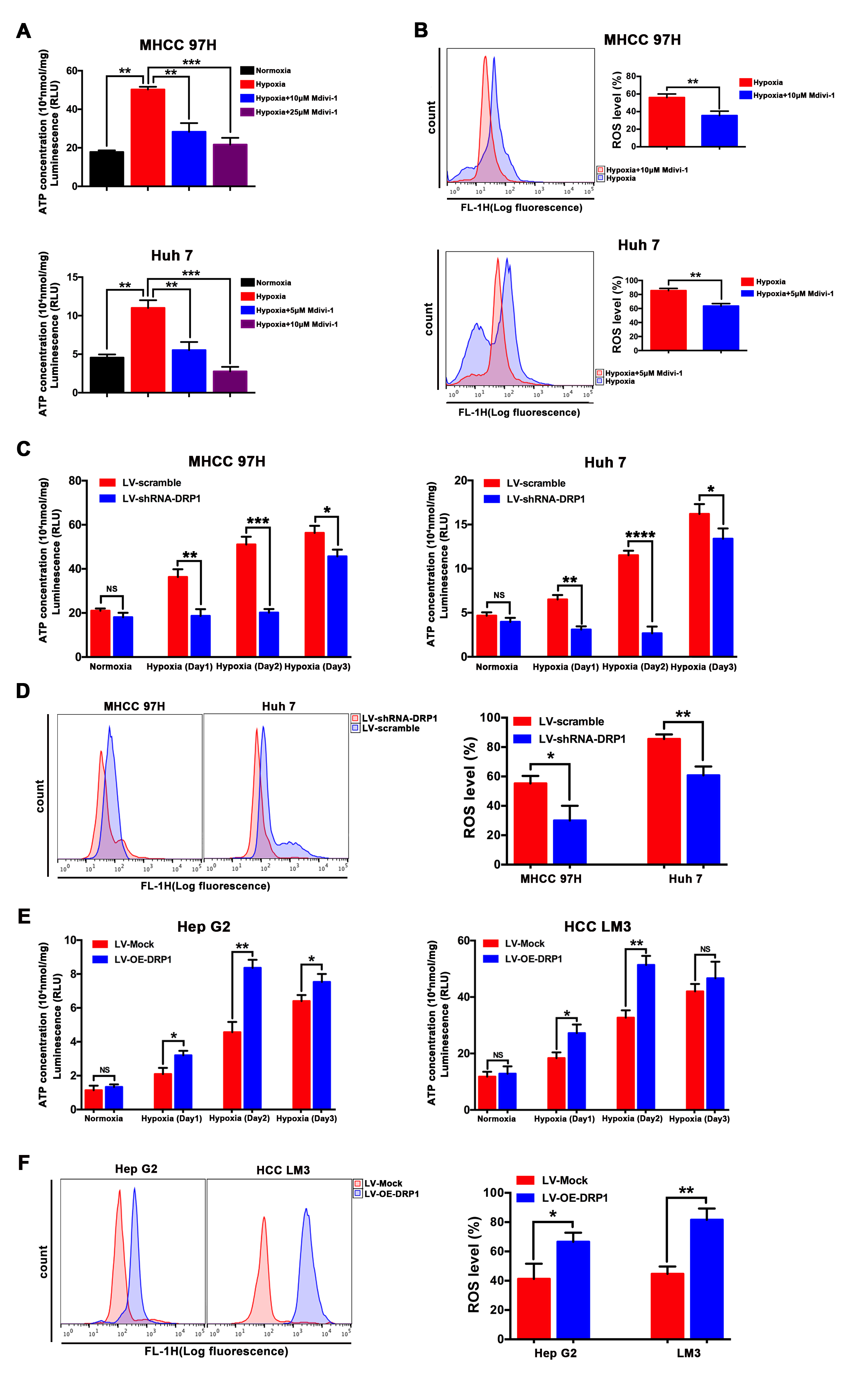

Supplement: Supplementary file 4 — Supplementary Figure S4 [file 41389_2020_251_MOESM4_ESM.tif]
