# Supplementary material for: Suppressing DRP1-mediated mitochondrial fission and mitophagy increases mitochondrial apoptosis of hepatocellular carcinoma cells in the setting of hypoxia
Source: Oncogenesis. 2020 Jul 13;9(7):67. doi: 10.1038/s41389-020-00251-5 (PMC7359348; doi:10.1038/s41389-020-00251-5)
Supplement: Supplementary file 5 — Supplemental Figure legends [file 41389_2020_251_MOESM5_ESM.docx]

**Supplemental Figure legends**

**Fig. S1** Hypoxia-treated HCC cells had a lower mitochondrial mass than the cells in normoxia as measured by COX IV staining (a marker to monitor the mitochondrial degradation in the process of mitophagy).

**Fig. S2 DRP1 inhibition by Mdivi‑1 led to cellular morphological alteration of hypoxia-surviving HCC cells.** (A) The viabilities of MHCC97H and Huh7 cells were detected by CCK-8 kit. (B) After Mdivi-1 treatment, HCC cells in hypoxia showed the morphological change from a typically long spindle-shaped morphology into round phenotype.

**Fig. S3 DRP1 expression in tumor tissues.** (A) Representative immunohistochemistry images of DRP1 expression in HCC tissues relative to the matched peritumor liver tissue. (B) The DRP1 expression level was compared between stage I–II and stage III–IV in HCC patients, as analyzed by immunohistochemistry.

**Fig. S4 DRP1 level had an impact on intracellular ATP content and ROS levels in hypoxia-surviving HCC cells.** (A, B) DRP1 inhibition by Mdivi‑1 influenced intracellular ATP content and ROS levels in HCC cells (MHCC97H and Huh7) during hypoxia. (C, D) DRP1 knockdown by lentiviral vectors expressing short hairpin RNA targeting DRP1 (LV-shRNA-DRP1) affected intracellular ATP content and ROS levels in HCC cells (MHCC97H and Huh7) in hypoxia. (E, F) DRP1 overactivation by lentiviral vectors expressing DRP1 (LV-OE-DRP1) changed intracellular ATP content and ROS levels in HCC cells (Hep G2 and HCCLM3) in hypoxia. **P* < 0.05, ***P* < 0.01, *** *P* < 0.001.
