# Supplementary material for: Suppressing DRP1-mediated mitochondrial fission and mitophagy increases mitochondrial apoptosis of hepatocellular carcinoma cells in the setting of hypoxia
Source: Oncogenesis. 2020 Jul 13;9(7):67. doi: 10.1038/s41389-020-00251-5 (PMC7359348; doi:10.1038/s41389-020-00251-5)
Supplement: Supplementary file 6 — Supplementary Table S1 [file 41389_2020_251_MOESM6_ESM.docx]

**Table S1** **Correlations between DRP1 expression and clinicopathological features in 100 patients with HCC**

| Variable | DRP1 expression | | *P*-value* |
| --- | --- | --- | --- |
|  | DRP1^Low^ | DRP1^High^ |  |
| Age, years |  |  | 1.000 |
| <55 | 24 | 24 |  |
| ≥55 | 26 | 26 |  |
| Gender |  |  | 0.790 |
| Female | 9 | 8 |  |
| Male | 41 | 42 |  |
| Tumor size (cm) |  |  | 0.045* |
| <5 | 29 | 19 | 0.424 |
| ≥5  Tumor number  1  ≥2 | 21  43  7 | 31  40  10 |  |
| Tumor encapsulation |  |  | 0.841 |
| Yes | 27 | 26 |  |
| No | 23 | 24 |  |
| Tumor Differentiation |  |  | 0.005* |
| Moderate/Well  Poor | 44  6 | 32  18 |  |
| TNM stage |  |  | 0.009* |
| I-II | 41 | 29 |  |
| III-IV | 9 | 21 |  |
| Recurrence |  |  | 0.005* |
| Yes | 21 | 35 |  |
| No | 29 | 15 |  |
| AFP |  |  | 0.142 |
| <20 | 21 | 14 |  |
| ≥20 | 29 | 36 |  |
|  |  |  |  |

Note: A chi-square test was used for comparing groups between low and high DRP1 expression. *p < 0.05 (2-tailed) was considered statistically significant.

Abbreviations: SD, standard deviation; AFP, alpha-fetoprotein.
