# Supplementary material for: Suppressing DRP1-mediated mitochondrial fission and mitophagy increases mitochondrial apoptosis of hepatocellular carcinoma cells in the setting of hypoxia
Source: Oncogenesis. 2020 Jul 13;9(7):67. doi: 10.1038/s41389-020-00251-5 (PMC7359348; doi:10.1038/s41389-020-00251-5)
Supplement: Supplementary file 7 — Supplementary Table S2 [file 41389_2020_251_MOESM7_ESM.docx]

**Table S2 Univariate and multivariate analysis of the factors associated with overall survival (OS) of HCC patients**

| Variable | Univariable OS | |  | | Multivariable OS | |
| --- | --- | --- | --- | --- | --- | --- |
|  | P-value* | HR (95% CI) | |  | P-value* | HR (95% CI) |
| Age, years  (<55 vs. ≥55) | 0.779 | 0.928(0.549-1.567) | |  | NA |  |
| Gender | 0.093 | 0.507(0.229-1.121) | |  | NA |  |
| (Female vs. Male)  Tumor size (cm)  (<5 vs. ≥5)  Tumor number  (1 vs. ≥2)  Tumor encapsulation  (Yes vs. No)  Tumor Differentiation | 0.014*  0.028*  0.995  0.433 | 1.961(1.144-3.362)  0.497(0.266-0.928)  1.002(0.593-1.693)  0.789(0.437-1.427) | |  | 0.021  0.358  NA  NA | 1.889(1.098-3.248)  0.727(0.369-1.433) |
| (Moderate/Well vs. Poor)  TNM stage | 0.190 | 0.693(0.400-1.199) | |  | NA |  |
| (I-II vs. III-IV)  AFP  (<20 vs. ≥20)  DRP1 staining | 0.539  0.001* | 0.839(0.478-1.470)  2.515(1.453-4.354) | |  | NA  0.001* | 2.449(1.412-4.249) |
| (low vs. high) |  |  | |  |  |  |

Note: *p < 0.05 (2-tailed) was considered statistically significant. p value was calculated using Cox proportional hazards regression.

Abbreviations: OS, overall survival; DRP1, Dynamin-related protein 1; HR, hazard ratio; CI, confidence interval; SD, standard deviation; NA, not considered in the multivariable model.
