# Supplementary material for: Suppressing DRP1-mediated mitochondrial fission and mitophagy increases mitochondrial apoptosis of hepatocellular carcinoma cells in the setting of hypoxia
Source: Oncogenesis. 2020 Jul 13;9(7):67. doi: 10.1038/s41389-020-00251-5 (PMC7359348; doi:10.1038/s41389-020-00251-5)
Supplement: Supplementary file 8 — Supplementary Experimental Procedures [file 41389_2020_251_MOESM8_ESM.docx]

**Supplementary Experimental Procedures**

**Materials and methods**

***Antibodies and reagents***

Mdivi-1 and DMSO were acquired from Sigma-Aldrich. This study used the primary antibodies against DRP1(Abcam, ab184247), phosphor-DRP1 (Ser616) (Cell Signaling Technology, USA, 3455), BNIP3 (Abcam, ab109362), LC3B (Cell Signaling Technology, USA, 3868), cleaved caspase-3 (Abcam, ab32042), Bax (Cell Signaling Technology, USA, 5023) and AIF (Cell Signaling Technology, USA, 5318), cytochrome C (Abcam, ab133504) and COX (Abcam, ab202554), TOM20（Santa-Cruz, USA）and GAPDH (Beyotime Biotechnology, China), whereas the secondary antibodies utilized were Alexa Fluor anti-mouse, rabbi IgG (Molecular Probe), and HRP-conjugated anti-mouse or anti-rabbit IgG (Cell Signaling).

***Cell culture and hypoxic treatment***

Human liver cells L02, HCC cells (PLC/PRF/5, HepG2 and SMMC7721) (Cell Bank of the Chinese Academy of Sciences, Shanghai, China), MHCC97H and HCCLM3 (Liver Cancer Institute, Fudan University, Shanghai, China), and Huh7 (Japanese Cancer Research Resources Bank) were cultured in the corresponding culture medium RPMI-1640 or Dulbecco's modified Eagle medium (Gibco) with 10% fetal bovine serum (Excell Bio, china) and 1% penicillin-streptomycin (Invitrogen). Cell cultures were done in a thermostatic incubator at 37°C with a humidified atmosphere of 95% air and 5% CO_2_. For hypoxic experiments, cells were incubated in an incubator with a humidified atmosphere of 1% O_2_, 5% CO_2_ and 94% N_2_. For Mdivi-1 treatment, HCC cells at 50% confluence were treated with DRP1 inhibitor Mdivi-1 (Sigma-Aldrich) for indicated periods.

***Knockdown or overexpression of DRP1***

Lentiviral vectors expressing short hairpin RNAs targeting DRP1 (LV-shRNA-DRP1) or a scramble RNA (LV-shRNA-scramble) were constructed by GeneChem Biotechnology (Shanghai, China). The lentiviruses were produced by transfecting 293T cells with the lentiviral vector and three lentiviral packaging vectors. HCC cells were plated in 6-well plates and transfected with the lentivirus (at a multiplicity of infection of 10) with 5μg/ml polybrene, based on manufacturer’s protocol. The knockdown efficiency of DRP1 was evaluated through qRT-qPCR and western blot analysis.

The pcDNA3.1 plasmid expressing DRP1 or a negative control was purchased from GeneChem Biotechnology (Shanghai, China). HCC cells at 50% confluence in 6-well plates were transfected with plasmids using the transfection reagent Lipofectamine 2000 (Invitrogen).

***Quantitative reverse-transcription polymerase chain reaction (qRT-PCR)***

The total RNA was extracted from cells or tissues using the TRIzol Reagent (Invitrogen), and then cDNA was synthesized from RNA using the Reverse Transcription Kit (Takara). Subsequently, cDNA was amplified using the Maxinma SYBR Green qPCR Master Mix (Thermo Scientific). The quantification of target genes was done with the 2^−ΔΔCt^ method using glyceraldehyde-3-phosphate dehydrogenase (GAPDH) for normalization. Melting curve analysis was carried out to assess the specificity of PCR products. The DRP1 primer has been used for real time PCR, Sequence 5’-3’: Forward, GCTGCTTCTGCTGAGGCTGATG; Reverse, TTGTGGACTGGCTGGCATAATTGG

***Cell viability***

The cell viability of HCC cells was determined using the Cell Counting Kit-8, in accordance with the manufacturer’s instruction (CCK-8, Yeasen, Shanghai, China).

***Flow cytometric analysis***

Apoptosis was analyzed using Alexa Fluor 488 Annexin V Kit (Invitrogen), in accordance with the manufacturer’s instructions. Briefly, the cells (1×10^6^ cells/ml) were harvested, washed with PBS and centrifuged at 1000 rpm for 5 min, and then the cell pellet was resuspended in an annexin-binding buffer and incubated with annexin V and PI working solution for 15 min at room temperature. Cell apoptosis was determined using FACS caliber Flow cytometer (BD Biosciences, San Jose, CA, USA) and FlowJo software (Tree Star, San Carlos, CA).

***Mitochondrial membrane potential***

JC-1 staining was used to assess the mitochondrial membrane potential was assessed. Cells were incubated with mitochondrial fluorescent JC-1 prober (1 μg/ml) and observed under a fluorescence microscope. JC-1 forms the aggregates at high transmembrane potential (intact mitochondria) and emits red fluorescence under confocal microscopy, whereas it maintains its monomeric form at low transmembrane potential and displays green fluorescence. The red and green fluorescence ratio of JC-1 indicates a mitochondrial membrane potential. The loss of mitochondrial membrane potential is indicated by the decreased red fluorescence and increased green fluorescence.

***Determination of intracellular ATP content***

The cells (2 × 10^5^/well) on 6-well plates were collected and the intracellular ATP content was measured using the ATP Determination Kit in accordance with the manufacturer’s instructions (Beyotime company).

***Reactive oxygen species (ROS) detection***

The intracellular ROS levels were measured using the Reactive Oxygen Species Assay Kit based on the manufacturer’s instructions (Beyotime company).

***HCC samples***

This study was approved by the Ethics Committee of Zhongshan Hospital of Fudan University (Shanghai, China) and written informed consent was obtained from each patient. Thirty pairs of frozen HCC tissues and matched nontumor liver tissues from 30 patients were subjected to qRT-PCR and western blot analysis. Another cohort of 100 patients who had histologically proven HCC and underwent complete surgical resection between January 2006 and December 2008 at Zhongshan Hospital of Fudan University (Shanghai, China) were also included. The HCC tissue microarray was constructed as previously described. Clinicopathological data were obtained from the electronic medical record database. Overall survival (OS) and recurrence-free survival (RFS) were defined as the time from surgery to the date of death or the date of recurrence.

***In Vivo Model***

Animal experiments were approved by the Committee on Animal Research of Zhongshan Hospital, Fudan University (Shanghai, China) and were performed conforming to the guidelines formulated by Shanghai Medical Experimental Animal Care Commission. Twelve BALB/c nude mice (male, 4-6 weeks old, weighting 18-20 g) were obtained from Shanghai SLAC Laboratory Animal Co., Ltd., China, and were maintained in animal cages in specific pathogen-free conditions with a 10‑h light/14‑h dark cycle, a constant temperature of 22-27˚C, and a relative humidity of 40-60 % and with an unlimited access to water and food. HCC cells were subjected to hypoxia for 3 days, followed by harvest and resuspension, and a suspension of 2x10^7^ Huh7 cells was subcutaneously injected into the right flank of each mouse. After 2 days, the mice were randomly divided into two groups: the control group (DMSO, n=6) and Mdivi-1 treatment group (intra-peritoneal injection of 50 mg/kg Mdivi-1, n=6). The treatment was administrated for 3 weeks. The mice were sacrificed at 48 h after the last treatment and tumor xenografts were harvested for further analyses.

***Statistical analysis***

Data are expressed as means ± standard deviations from three independent experiments and are analyzed using SPSS software (21.0; SPSS, Inc, Chicago, IL). Continuous variables between two groups or among three groups were compared using the unpaired Student’s *t*-test or one-way analysis of variance (Bonferroni post hoc test) as appropriate, and categorical variables were compared using the Chi-square test or Fisher’s exact test as appropriate. OS and RFS curves were generated using the Kaplan-Meier method and were compared using the log-rank test. A two-sided *P*-value of <0.05 was considered statistically significant.
